# Characterization of the high-pressure and high-temperature phase diagram and equation of state of chromium

**DOI:** 10.1038/s41598-022-10523-2

**Published:** 2022-04-25

**Authors:** Simone Anzellini, Daniel Errandonea, Leonid Burakovsky, John E. Proctor, Robin Turnbull, Christine M. Beavers

**Affiliations:** 1 Diamond Light Source Ltd., Harwell Science and Innovation Campus, Diamond House, Didcot, OX11 0DE UK; 2 Departamento de Física Aplicada-Instituto de Ciencia de Materiales, Matter at High Pressure (MALTA) Consolider Team, Universidad de Valencia, Edificio de Investigación, C/Dr. Moliner 50, Burjassot, 46100 Valencia, Spain; 3 Theoretical Division, Los Alamos National Laboratory, Los Alamos, New Mexico 87545 USA; 4 Materials and Physics Research Group, School of Science, Engineering and Environment, University of Salford, Manchester, M5 4WT UK

**Keywords:** Materials science, Condensed-matter physics

## Abstract

The high-pressure and high-temperature phase diagram of chromium has been investigated both experimentally (in situ), using a laser-heated diamond-anvil cell technique coupled with synchrotron powder X-ray diffraction, and theoretically, using ab initio density-functional theory simulations. In the pressure–temperature range covered experimentally (up to 90 GPa and 4500 K, respectively) only the solid body-centred-cubic and liquid phases of chromium have been observed. Experiments and computer calculations give melting curves in agreement with each other that can both be described by the Simon–Glatzel equation $T_{m}(P) = 2136K (1 + P/25.9)^{0.41}$. In addition, a quasi-hydrostatic equation of state at ambient temperature has been experimentally characterized up to 131 GPa and compared with the present simulations. Both methods give very similar third-order Birch–Murnaghan equations of state with bulk moduli of 182–185 GPa and respective pressure derivatives of 4.74–5.15. According to the present calculations, the obtained melting curve and equation of state are valid up to at least 815 GPa, at which pressure the melting temperature is 9310 K. Finally, from the obtained results, it was possible to determine a thermal equation of state of chromium valid up to 65 GPa and 2100 K.

## Introduction

Transition metals are the elements in the *d*-block of the periodic table which exhibit partially filled *d*-electron shells. They are typically characterized by their high densities, cohesive energies, bulk moduli, and melting temperatures. Many of them are refractory metals. Due to their typically high melting temperatures ($T_m$) and hardness, the technological applications and economic importance of these elements (and their alloys) is immense. From an engineering point of view, the need to control the properties of transition metal alloys has stimulated theoretical and experimental studies on the phase relations of the pure elements. These metals have also been the focus of fundamental research, mainly for understanding the influence of *d*-electrons on the properties of the elements. Additionally, the study of transition metals under high-pressure (*HP*) and high-temperature (*HT*) is extremely relevant for geophysics and planetary sciences because these metals represent the main constituents of planetary cores (e.g. Fe and Ni in the Earth)^1^. In particular, over the past two decades the characterization of the melting curves of transition metals has attracted the interest of many researchers around the globe. This has been mainly due to the discrepancies observed in the melting temperatures determined via different experimental (static and dynamic) and computational techniques. In particular, considerable effort has been devoted to trying to solve the discrepancies observed in the extreme cases of Fe^2–11^, Ta^12–14^, Mo^15–17^ and Pt^18–21^. The melting curves of other transition metals, like Nb, Ir, and V have also been studied recently^22–24^.

In contrast with other transition metals, chromium (Cr) is one of the elements of this group less studied under *HP*–*HT* conditions. With an electronic configuration [Ar]3d$^5$4s$^1$, Cr belongs to the family of the 3*d* transition metals (which also includes Mn, Fe, Co and Ni). Cr is a hard and brittle element, highly valued for its high resistance to corrosion. For this reason, it is used as the main additive in stainless steel and in other metallurgic processes. Under ambient conditions, Cr exhibits a body-centred-cubic (*bcc*) structure. At low *T* and *HP* the *bcc* structure of Cr is slightly modified by two first-order magnetic phase transitions^25^. From 0 to 123 K, Cr is antiferromagnetic (AF) with a small tetragonal distortion of the *bcc* structure. Whereas, from 123 to 311 K, it is AF with a small orthorhombic distortion of the *bcc* structure. These phase transitions have been measured to 0.8 GPa, however the lattice distortion of these phases is too small to be detected by X-ray diffraction (XRD)^26,27^. The physical reasons for the ground state of Cr being AF have been discussed in several papers. For example, Asano and Yamashita^28^, provide arguments to discuss the ferromagnetic (FM) nature of *bcc*-Fe, whilst both *bcc*-Mn and *bcc*-Cr are AF. Above temperatures of 311 K, Cr is paramagnetic (PM) and exhibits a *bcc* structure. According to theoretical calculations, Cr is expected to exhibit a *P*-induced polymorphism similar to both Mo and W, its partners in the periodic table, the *HP* crystal structure of which is double-hexagonal-close-packed (*dhcp*)^29^. Indeed, a pressure-induced phase transition from *bcc* to hexagonal-close-packed (*hcp*) has been predicted for Cr, with the transition *P* spanning the range 7–12.5 Mbar^30^.

The first static compression study of Cr at room-temperature (*RT*) was performed only up to 10 GPa^31,32^. More recent studies on Cr-hydrates reported synchrotron XRD measurements of pure Cr compressed to 40 GPa^33,34^, thereby providing information on the Cr bulk modulus. None of these studies have shown any sign of *P*-induced solid-solid phase transitions. Similar conclusions have been obtained from shock-compression studies up to 140 GPa^35^. At ambient pressure Cr melts at 2136 K^36^. In contrast to most of the other transition metals, the behaviour of Cr at *HP*–*HT* is still mostly unknown. The only reported melting curve of Cr was obtained two decades ago by Errandonea et al.^37^ (along with the melting curves of V and other transition metals) up to 60 GPa and 2600 K. The Cr melting curve was determined from an experiment performed in a single-sided laser-heated (LH) diamond-anvil cell (DAC), using the speckle technique to detect melting. In this technique, a direct observation of movements on the sample surface, assigned to the transformation of solid Cr into liquid Cr, was used as a melting diagnostic without any other structural characterization. Just like in other cases obtained using the same melting diagnostic^2,13,38^, the reported melting curve appeared to belong to the so called “low” melting curves^39,40^ which virtually flattens out at $P\sim$ 1 Mbar and for which the determined $T_m$ is much lower than that extracted from shock compression data. Furthermore, the reported Cr melting curve appeared to virtually coincide with the melting curve of V. The latter, after recently being very thoroughly re-measured using a state-of-the-art technique^24^ has been shown to have been originally underestimated, whereby the most recently determined melting curve belongs to the so-called “high” melting curves^39,40^, in agreement with shock measurements. To the best of our knowledge, there are no computational studies yet reported of the melting curve of Cr.

From the results summarized above, it becomes clear that a proper characterization of the phase diagram of Cr at *HP*–*HT* is still lacking. For this purpose, we herein report a combination of in situ synchrotron XRD studies on Cr up to 131 GPa and 4500 K and density-functional theory (DFT) calculations aiming to characterize the phase diagram, melting curve, and thermal equation of state (EoS) of Cr.

## Results and discussion

### Phase diagram

Eight *HP*–*HT* ramps were performed using the LH-system of the I15 beamline^41^ at Diamond Light Source, investigating a *P*–*T*-range between 10–90 GPa and 300–4500 K, respectively. The in situ XRD analysis revealed only the presence of the KBr-B2 phase, (which acted as pressure medium and thermal insulator between sample and diamonds), and the *bcc* and the liquid phases of Cr in the investigated *P*–*T* range. Furthermore, the obtained data did not show any distortion of the *bcc* structure of Cr or any evidence of the occurrence of any chemical reactions leading to the formation of oxides or carbides e.g. Cr$_3$C$_2$^42^ or Cr$_2$O$_3$^43^.

**Figure 1:**
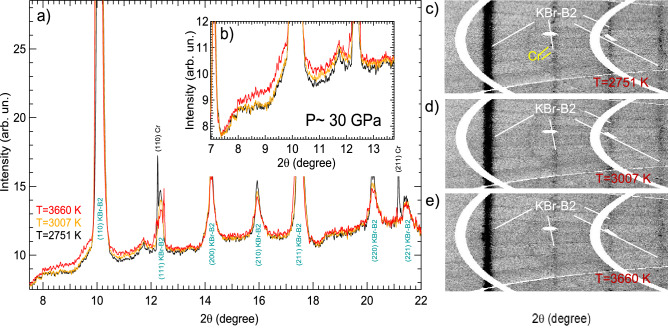
(**a**) Integrated XRD patterns at different *T* around 30 GPa and (**b**) corresponding zoomed region around the XRD diffuse signal. The onset of melting, characterized by the first appearance of the diffuse signal, is observed at 3007 K (orange pattern). The diffuse scattering increases with the rising *T*, showing its maximum at 3660 K (red pattern). (**c**–**e**) unwrapped raw XRD images showing the textural evolution of a Cr sample embedded in KBr pressure transmitting medium at around 30 GPa. The signals arising from the different components are highlighted with different colours: yellow for the Cr and white for the KBr-B2. *T* is also indicated in red.

**Figure 2:**
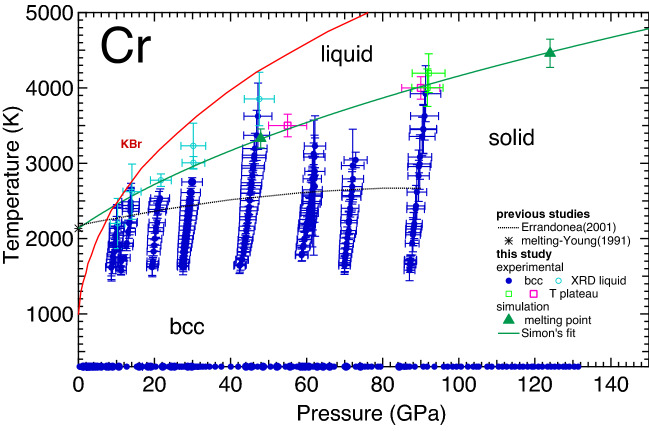
Phase diagram of Cr as obtained in the present study both experimentally (solid and empty circles and empty squares) and with first principle calculation (solid triangles and solid green line) and a previous study based on the speckle technique^37^ (dashed black line). The melting line of KBr as reported in Briggs et al.^44^ is also represented as a continuous red line for reference.

During each experimental run, it was possible to study the textural evolution of the sample (and the insulating material) as a function of *P*–*T*. Figure 1c–e shows an example of the textural evolution observed at around 30 GPa from ambient *T* up to 3660 K. In particular, the ambient *T* texture of KBr (highlighted in white in the figure) does not change up to 3007 K, however, at 3660 K thermal effects are clearly evident by the reduction of the full width at half maximum (FWHM) of the (110) reflection of KBr-B2 and the general reduction of intensity of the other of KBr reflections. The signal from Cr (highlighted in yellow in the figure) shows the texture of a highly oriented powder at 2751 K. At 3007 K, it is not possible to observe any Bragg peaks from crystalline Cr. The disappearance of the two Bragg peaks of *bcc* Cr, that can be observed due to the angular aperture of the set-up, is probably caused by the melting of Cr. A better visualization of this behaviour can be obtained from the corresponding integrated signals of the patterns reported in Fig. 1a,b together with the patterns obtained at *T* just below and above 3007 K. In particular, it is possible to observe how, at this *T*, the (110) and (211) reflections of *bcc*-Cr disappear whilst a diffuse XRD signal appears close to where the previous (110) reflection was located. Such a phenomenon is usually correlated to melting of transition metals^18,22–24^, therefore it is very reasonable to correlate it to the formation of liquid Cr. A further *T* increase causes the appearance of an additional (and more intense) diffuse signal, the center of which is shifted towards lower angles. We believe that this additional diffuse signal is caused by the melting of KBr. This observation is in agreement with the melting curve obtained by Briggs et al.^44^ which is plotted in Fig. 2 as a continuous red line. Figure 2 reports the phase diagram of Cr as obtained in the present experiment, compared with previous experimental data. A diffuse signal in the integrated XRD pattern was observed in all the *HT* ramps performed between 10 GPa and 50 GPa which was considered to be the appearance of liquid Cr (reported in the figure by empty light blue circles). However, we believe that the diffuse signal observed at 10 GPa actually originates from the melting of KBr (as evidenced by the proximity of the reported KBr melting curve) and that this is the reason for why the reported $T_{m}$ is lower than predicted by calculations and why it is comparable to the melting temperature of Cr at ambient pressure.

During the *HT* ramps performed around 60 GPa and 70 GPa and *T* higher than 3200 K, we noticed a misalignment in the optics of the heating system, causing *T* and XRD to be collected from different regions of the sample. This was also evidenced by the absence of thermal expansion in the lattice parameter of Cr. For this reason, all the data collected at *T* higher than 3200 K in these two ramps have been discarded as unreliable. Finally, we did not observed any diffuse signal in the ramp performed around 90 GPa, however, a plateau appeared in the plot of *T* vs laser power at around 4000 K (reported as empty light green squares in Fig. 2) and we have decided to underline it as a possible melting point as has been underlined in previous LH-DAC experiments on other metals^18,23,38,45,46^. The corresponding $T_m$ is in agreement with our computer simulations.

Additionally, off-line experiments were performed on Cr in order to search for temperature plateaus like the one observed in XRD diffraction experiments at 90 GPa. For this purpose, the samples were prepared following a similar procedure to the one used for the aforementioned experiments performed at Diamond Light Source, but instead using MgO as the insulating material (to exclude any effects caused by the melting of KBr). As for the experiments performed on beamline I15, *T* was measured via spectral-radiometry, whereas *P* was measured from ruby fluorescence method at 300 K. The thermal *P* was estimated by comparison with similar data from a previous XRD experiment performed using MgO as the insulating material^22^. The temperatures obtained for the plateaus in these ramps are (3500 ± 150) K and (4000 ± 150) K at (55 ± 5) GPa and (90 ± 5) GPa, respectively. The plateaus are reported in Fig. 2 as empty pink squares. Temperature plateaus are exhibited when a material undergoes a phase transition; for instance, melting or a solid-solid phase transition. In the present case of Cr, the two data points corresponding to temperature plateaus detected in off-line experiments correlate well with the calculated melting curve and with the melting curve extrapolated from XRD measurements described above. Therefore, we have tentatively assigned them to the melting of Cr.

The results obtained from the present computer simulations are reported in Fig. 2 as solid green lines and green triangles, and they are also summarized in Table 1. Specifically, six *bcc*-Cr melting points have been calculated assuming both a non-magnetic (NM) configuration and two assuming a paramagnetic (PM) *bcc*-Cr one. From the obtained results we have concluded that, at a given pressure, the calculated melting temperatures of both PM-Cr and NM-Cr are virtually identical. Our theoretical melting curve of bcc-Cr is based on the six points for NM-Cr listed in Table 1. For each of the melting points, ten NVE (fixed total number of atoms N, system volume *V*, and total energy E; *V* corresponds to one of the six densities from Table 1) runs of 10,000–20,000 time steps of 1.0 fs each were performed, with an increment of the initial *T* of 125 K for the 1st, 250 K for the 2nd, 375 K for the 3rd and 4th, and 500 K for the 5th and 6th $T_m$. The corresponding error in $T_m$ is half of the increment of the initial *T*^47^ so that it does not exceed $\sim$ 4$\%$ in any case. The *P* errors are negligibly small: ${\mathop {\sim }\limits ^{<}}0.5$ GPa for the first point, and 1–2 GPa for the remaining five. Hence, our melting results on *bcc*-Cr are very accurate.

**Table 1:** The six ab initio melting points of *bcc*-Cr, $(P_m,\,T_m\pm \Delta T_m),$ obtained from the *Z* method implemented with VASP.

Lattice constant (Å)	$P_m$ (GPa)	$T_m$ (K)	$\Delta$ $T_m$ (K)
3.00	$-$ 5.2	1950	62.5
2.80	47.9	3330	125.0
2.65	124	4460	187.5
2.50	266	5840	187.5
2.35	526	7720	250.0
2.25	815	9310	250.0

**Figure 3:**
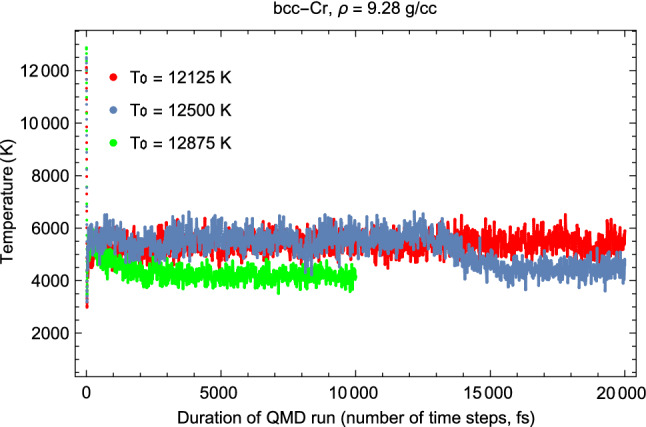
Time evolution of temperature in three QMD runs with initial temperatures (T$_0$) each separated by 375 K. The middle run is the melting run during which *T* decreases from $\sim$ 5500 K for the superheated state to 4460 K for the liquid at the corresponding melting point.

**Figure 4:**
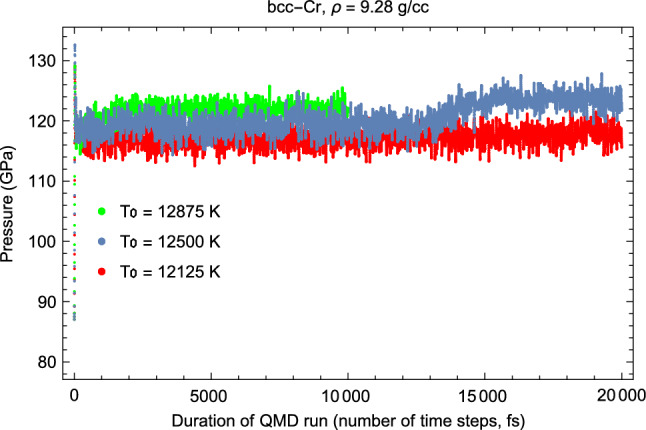
Time evolution of pressure in three QMD runs with initial temperatures (T$_0$) each separated by 375 K. During melting P increases from $\sim$ 120 GPa for the superheated state to 124 GPa for the liquid at the corresponding melting point.

Figures 3 and 4 offer examples of the present *Z* method for melting simulations. They correspond to the third of the six calculated melting points summarized in Table 1. These figures provide the time evolution of *T* and *P*, respectively, during the corresponding computer simulation runs. During the $T_0=12,125$ K run the system remains a super-heated solid: both the average *T* and *P* stay virtually the same during the 20 ps duration of run time. The $T_0=12,500$ K run is the melting run during which melting occurs: it starts after $\sim 13$ ps of run time, and the melting process takes about 3 ps. It results in the decrease of average *T* from $\sim 5500$ to 4460 K, and the corresponding increase of average *P* from $\sim 118$ to 124 GPa. This happens in the calculations because the total energy, $E\sim k_B\,T\,+\,P\,V,$ is conserved and *V* is fixed. For the same reason, Figs. 3 and 4 are “mirror images” of each other. In the run with highest $T_0=$ 12,875 K, the melting starts after only 1 ps of run time. For a sufficiently high initial *T* the system melts virtually immediately.

**Figure 5:**
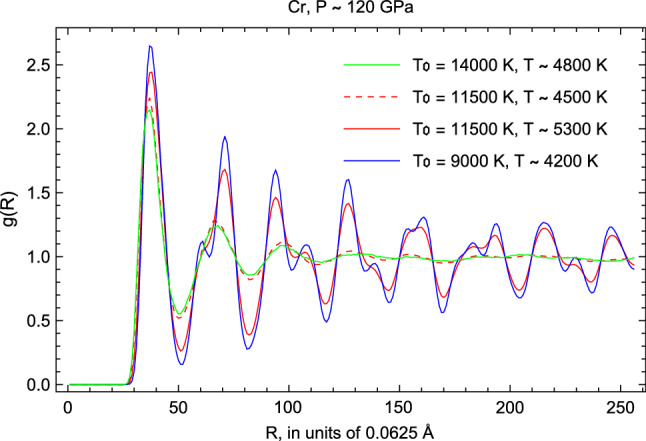
Radial distribution functions (RDFs) of equilibrium states of Cr in QMD Z-method runs at P $\sim$ 120 GPa. The corresponding initial and equilibrium temperatures are indicated as $T_{0}$ and *T*, respectively.

Unambiguous evidence of melting can be obtained from the calculated radial distribution functions (RDFs) of the equilibrium states of Cr which are shown in Fig. 5. As an illustration we provide in Fig. 5, the calculated RDFs of equilibrium states of Cr obtained from the QMD Z-method runs at P $\sim$ 120 GPa . A change from solid-like to liquid-like behaviour is clearly seen in the melting run at $T_0$ = 11,500 K during which the heights of the first and second peaks, and the depth of the first trough, are reduced. The higher-order peaks of superheated solid at T $\sim$ 5300 K before melting (first 13 ps of running time, red solid like in Fig. 5) become smoothed out at T $\sim$ 4500 K (last 4 ps of running time, red dashed line in Fig. 5) indicating liquid behaviour. RDFs of solid Cr at $\sim$ 300 K below the melting temperature, and of liquid Cr at $\sim$ 300 K above the melting temperature are added for better comparison with the data from the melting run.

From the analysis of the data in Fig. 2 it is possible to state that the present experimental and theoretical melting lines are in good agreement (to within the experimental errors). The corresponding melting line can be described with a Simon-Glatzel equation^1^ with the following expression $T_{m}(P) = 2136K (1 + P/25.9)^{0.41}$, where 2136 K is the $T_m$ at ambient *P* as reported in Young^36^. This equation provides a good estimate of the melting line of Cr and describes properly both the experiments and the melting temperatures calculated up to 815 GPa (9310 K). Notably the melting curve of Cr runs parallel to that of V^24^, the neighboring element in the periodic table. In particular, at low pressure the melting slope for Cr is $dT_{m}/dP$ = 34.9 K/GPa, while in V it is 32.6 K/GPa.

Let us now compare the determined melting slope to that given by the Clausius–Clapeyron (CC) formula $dT_m(P)/dP|_{P=0}=T_m(0)\cdot \Delta V_m/\Delta H_m$ with the experimental input. According to Stankus^48^, the ambient melting density of liquid Cr is $\rho _L=6.170$ g/cm$^3.$ This, along with the density of the solid before melting, $\rho _S=6.575$ g/cm$^3$, leads to a volume change upon melting of $\Delta V_m=0.519$ cm$^3$/mol. According to another set of thermal expansion data on Cr^49^, $\rho _L=6.156$ g/cm$^3$ and $\rho _S=6.509$ g/cm$^3,$ so that $\Delta V_m=0.458$ cm$^3$/mol. Therefore, we consider their average $\Delta V_m=0.49\pm 0.03$ cm$^3$/mol. Taking the melting enthalpy from the literature, $\Delta H_m=29\pm 1$ kJ/mol^50,51^, the CC formula gives $dT_m(P)/ dP|_{P=0}=36.1\pm 2.5,$ K/GPa, which is in good agreement with both the present theoretical and experimental melting results. Another theoretical value of the initial slope, 33.5 K/GPa^52^, is also consistent with the CC formula. We note that the use of $\Delta H_m=21$ kJ/mol from^53^ would result in a value of $dT_m(P)/dP|_{P=0}$ about 50% larger, which would contradict our experimental data. This strongly suggests that the assessment of Ref.^53^ is incorrect, including their value of $T_m(0)=2180$ K.

Finally, from the present in situ and computational characterization, it is possible to conclude that the direct observation of movement on the sample surface as a melting diagnostic^37^ (dashed black line in Fig.2) actually underestimates the melting curve of Cr. In particular, the underestimation is as large as 8$\%$ at 10 GPa, and 34$\%$ at 90 GPa, compared to the current melting curve. This confirms the unreliability of the direct observation of movement on the sample surface (also known as the ‘speckle technique’) as melting diagnostic. In fact, there are elements for which the melting lines obtained by this technique and by in situ XRD show good agreement (e.g Ca^54^, Pt^18^, Al^55^ and Cu^56^). However, in most cases, the melting line obtained via the speckle technique underestimates the true melting line of the studied elements (e.g Fe^2^, Ni^38^, Ta^13^, Mo^16^, etc.), sometimes by thousands of K, and the present work shows that this is the case for Cr.

### Cold compression curve

A cold compression run on Cr was carried out at *RT* at the extreme conditions beamline (I15) of Diamond Light Source under quasi-hydrostatic conditions (using Helium (He) as the pressure transmitting medium). The experiment was performed up to 131 GPa and the obtained Cr unit-cell parameters at each pressure are reported in Table 2. Cr maintains its *bcc* structure in the entire investigated *P* range. When analyzing the XRD patterns of Cr, special attention was taken to identify the possible occurrence of a *P*-induced rhombohedral distortion, as observed in V at 60 GPa^24^. In V, this distortion causes a splitting of the (211) reflection. In the present study we observed that such a splitting does not occur, indicating that the cubic-rhombohedral transformation does not take place in Cr up to at least 131 GPa.

**Figure 6:**
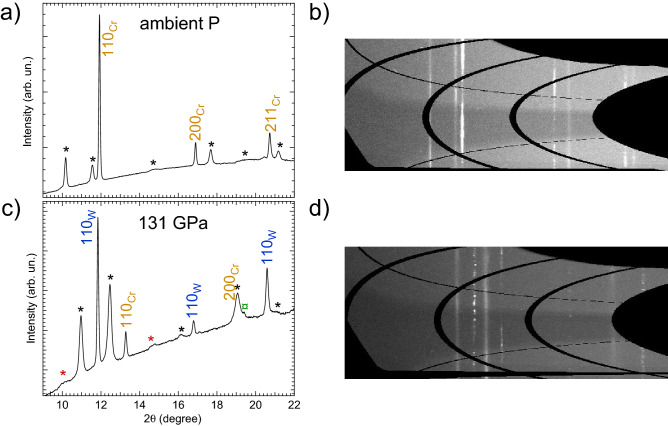
Integrated powder XRD patterns of Cr at the lowest (**a**) and the highest (**b**) *P* reached in the present experiment and their corresponding image plates (**c**,**d**), respectively.The different coloured asterisks are indicating the peaks from the Re gasket. The empty green symbol labels the peak obtained from solid He. The pattern in (**a**) was collected before the He loading and the DAC was positioned to obtain only the signal from Cr. In (**c**), the DAC was positioned to obtain signal from both Cr and W (the pressure standard) simultaneously.

Figure 6 shows the azimuthally-unwrapped raw XRD images and corresponding integrated patterns obtained at the lowest and highest *P* reached in this experiment. From an analysis of the unwrapped images, it is possible to observe how the texture of Cr evolves from a powder-like signal, with some preferred orientations at ambient conditions, into a more speckled (highly oriented) signal at 131 GPa. The pattern in Fig. 6a was collected before loading He into the DAC. In this way it was possible to check the quality of the Cr sample before starting the actual experiment and to obtain a perfect V$_0$ to use for subsequent fitting of the EoS. In both cases it is possible to observe the presence of Re signal (labelled with asterisks in Fig. 6a,c) due to the small diameter of the sample chamber in the Re gasket and the divergence of the X-ray focusing on I15’s micro-focus station, whereby the tails of the X-rays beam diffract through the edge of the Re pressure chamber. At *HP* it is also possible to observe a splitting of the Re peaks into two groups, one at the same *P* as the sample (black asterisk in Fig. 6c), according to the Re EoS of Anzellini et al.^57^ and one at lower *P* (red asterisk in Fig. 6c). Such a behaviour has been also observed in recent experiments performed with toroidal DACs^58^ and it is probably due to a combination of effects caused by the *P*-induced deformation of the gasket and the presence of the above-discussed X-ray tails. In fact, due to the corresponding shrinkage of the high pressure chamber, at *HP* the X-rays can simultaneously probe both the Re gasket in contact with He (same pressure as the sample) and the Re gasket squeezed directly between the anvils. In Fig.6c it is also possible to observe the presence of a peak around $2\theta = 19^{\circ }$ due to solid He. This peak appeared for the first time around 26 GPa and its behaviour under compression agreed perfectly with the He EoS of Loubeyre et al.^59^. When possible, the (110), (200) and (211) reflections of *bcc* Cr were used in the Pawley method to obtain the Cr lattice parameter. However, with increasing pressure, the (200) and (211) reflections of Cr started overlapping with peaks from Re, leaving the Cr (110) as the only usable reflection. Thanks to the cubic nature of Cr, this overlap of reflections did not create problems for the present characterization.

A qualitative analysis of the hydrostatic conditions of the sample was performed by comparing the *d*-spacing of Cr measured at the highest *P* reached in the present experiment with the theoretical pressure obtained using the same lattice parameters. The observed deviation of 0.01 $\%$ is well inside the power resolution of I15 beamline obtained from a similar analysis of the signal from the CeO$_2$ standard at ambient *P*. That means that any possible deviation from hydrostaticity is too small to be detected experimentally. Therefore, we can consider the present compression curve as quasi-hydrostatic.

The present DFT calculations show that the (*P* = 0, *T* = 0) ground state corresponds to a *bcc*-Cr with a density of 7.229 g/cm$^3$ (a lattice constant of 2.88 Å or an atomic volume of 23.888 Å$^3$), and a molar volume of 7.193 cm$^3$, in excellent agreement with 7.2 cm$^3$ from the present and previous experiments^60^. At this density, the NM *bcc*-Cr is higher in energy by $\sim$ 20 meV/atom, *i*.*e*., the ground state of *bcc*-Cr is correctly predicted to be antiferromagnetic (AF). However, the calculated magnetic moment per atom (MMA), $\sim$ 1.1 $\mu _{\mathrm{B}}$ is somewhat larger than the experimental value of 0.62 $\mu _{\mathrm{B}}$^61^ which is a typical discrepancy for this type of theoretical calculations^61,62^. The MMA depends almost linearly on the *bcc*-Cr lattice constant, *a* :  $\mu (a)\approx 1.1\,+\,14\,(a-2.88),$ and it becomes zero at $a\approx 2.8$ Å (a density of 7.87 g/cm$^3$). The corresponding *P* is $\sim 22$ GPa. This is higher than $a\approx 2.84$ Å at $P\approx 10$ GPa where the experimental Néel *T*,  as $T=T(a)$ and *T* = *T*(*P*), becomes zero^63^, but it is consistent with another theoretical work^62^ which demonstrates that the calculated magnetic moment is considerably suppressed at a pressure of $\sim 20$ GPa. At $P {\ge } 20$ GPa the EOS of AF *bcc*-Cr merges into that of non-magnetic (NM) *bcc*-Cr, so that the two EoSs are virtually identical. Both AF *bcc*-Cr below 20 GPa and NM *bcc*-Cr above 20 GPa combined are described by a single third-order Birch–Murnaghan EoS. The EoS parameters are summarized in Table 2. This EOS is expected to be reliable to $\sim 2$ TPa.

We note that paramagnetic (PM) Cr is not explicitly included in the above EOS. To check the possible influence of this exclusion on the present results, we calculated the EoS of PM *bcc*-Cr using the GGA+U scheme with spin-orbit coupling suggested for the calculation of the properties of paramagnetic materials^64^. We used the Dudarev approach^65^ in which the parameters *U* and *J* do not enter separately since only their difference $U-J$ is meaningful. The value of $U-J=4.5$ eV (actually, $U=4.5$ eV and $J=0$) comes from^66^. We found that the EoS of PM *bcc*-Cr virtually coincides with that of NM *bcc*-Cr, because the difference in the two values of *P* at the same density becomes negligibly small as density increases. Since their EoSs are virtually identical, too, it does not matter what structure of *bcc*-Cr, paramagnetic or nonmagnetic, is considered.

**Table 2:** The unit-cell parameters of Cr at ambient *T* as a function of *P*.

a$_W$ (Å)	P$_W$ (GPa)	a$_{Cr}$ (Å)	V$_{Cr}$ (Å$^3$)	a$_W$ (Å)	P$_W$ (GPa)	a$_{Cr}$ (Å)	V$_{Cr}$(Å$^3$)	a$_W$ (Å)	P$_W$ (GPa)	a$_{Cr}$ (Å)	V$_{Cr}$ (Å$^3$)
3.167	0.00	2.886	24.03	3.042	45.97	2.730	20.34	2.926	114.75	2.612	17.82
3.165	0.40	2.884	24.00	3.039	47.09	2.726	20.27	2.925	116.00	2.610	17.78
3.165	0.32	2.884	23.99	3.036	48.86	2.723	20.18	2.923	116.89	2.609	17.75
3.165	0.38	2.884	23.99	3.032	50.50	2.719	20.10	2.922	118.11	2.608	17.73
3.165	0.38	2.884	23.98	3.028	52.65	2.714	19.99	2.920	119.22	2.606	17.70
3.165	0.47	2.884	23.98	3.024	54.44	2.711	19.92	2.920	119.73	2.605	17.68
3.165	0.51	2.884	23.98	3.021	56.35	2.707	19.85	2.918	120.94	2.603	17.64
3.164	0.65	2.884	23.98	3.019	57.48	2.704	19.77	2.917	121.94	2.602	17.62
3.164	0.66	2.884	23.98	3.014	59.77	2.700	19.68	2.915	123.36	2.601	17.59
3.163	0.94	2.883	23.96	3.011	61.66	2.697	19.62	2.914	124.25	2.600	17.57
3.161	1.38	2.880	23.89	3.008	62.95	2.695	19.57	2.912	125.21	2.598	17.53
3.149	5.18	2.862	23.44	3.007	63.55	2.693	19.54	2.911	126.29	2.597	17.51
3.125	12.71	2.832	22.72	3.003	65.60	2.688	19.41	2.909	127.49	2.595	17.48
3.117	15.34	2.822	22.48	3.002	66.34	2.686	19.37	2.909	128.17	2.594	17.46
3.107	18.86	2.809	22.16	3.000	67.19	2.684	19.34	2.907	129.07	2.593	17.43
3.106	19.49	2.806	22.10	2.995	69.94	2.680	19.26	2.906	130.34	2.592	17.41
3.103	20.32	2.803	22.03	2.995	70.08	2.679	19.23	2.905	131.39	2.590	17.38
3.097	22.63	2.797	21.88	2.994	70.61	2.678	19.20				
3.094	23.80	2.794	21.81	2.990	72.92	2.675	19.14				
3.091	24.87	2.791	21.75	2.988	74.05	2.673	19.09				
3.090	25.51	2.789	21.70	2.986	75.29	2.671	19.05				
3.089	25.62	2.787	21.66	2.985	76.12	2.669	19.02				
3.089	25.82	2.786	21.62	2.983	77.09	2.668	18.98				
3.087	26.57	2.785	21.59	2.980	78.74	2.665	18.94				
3.087	26.60	2.783	21.56	2.979	79.40	2.664	18.91				
3.085	27.15	2.782	21.54	2.969	85.41	2.654	18.70				
3.085	27.10	2.781	21.51	2.969	85.67	2.654	18.69				
3.084	27.68	2.780	21.49	2.969	85.70	2.654	18.69				
3.083	28.24	2.779	21.46	2.969	85.92	2.653	18.68				
3.082	28.48	2.777	21.42	2.968	86.09	2.653	18.68				
3.082	28.46	2.777	21.42	2.968	86.40	2.653	18.67				
3.081	29.03	2.775	21.36	2.967	87.07	2.651	18.64				
3.077	30.64	2.772	21.30	2.965	88.41	2.649	18.59				
3.077	30.54	2.771	21.29	2.961	90.68	2.647	18.54				
3.077	30.48	2.771	21.28	2.960	91.68	2.645	18.51				
3.077	30.69	2.771	21.27	2.957	93.60	2.643	18.45				
3.075	31.22	2.770	21.26	2.953	96.04	2.640	18.40				
3.075	31.15	2.770	21.25	2.951	97.09	2.638	18.36				
3.075	31.43	2.769	21.24	2.949	98.36	2.636	18.31				
3.074	31.68	2.769	21.22	2.948	99.39	2.634	18.27				
3.074	31.54	2.768	21.21	2.946	100.6	2.632	18.23				
3.075	31.40	2.768	21.20	2.944	102.37	2.629	18.18				
3.073	32.17	2.767	21.17	2.942	103.79	2.627	18.14				
3.072	32.74	2.765	21.14	2.940	104.66	2.626	18.11				
3.070	33.48	2.763	21.09	2.940	105.22	2.625	18.09				
3.068	34.46	2.760	21.02	2.936	107.49	2.622	18.03				
3.064	35.95	2.755	20.91	2.935	108.64	2.620	17.99				
3.060	37.59	2.751	20.83	2.932	110.45	2.617	17.92				
3.055	40.06	2.745	20.69	2.931	111.50	2.616	17.91				
3.050	42.00	2.740	20.57	2.929	112.58	2.615	17.88				
3.045	44.40	2.734	20.45	2.927	113.87	2.613	17.84				

All values are obtained using He as pressure transmitting medium. The lattice parameters of W, used for the *P*^67^ measurements, are also reported. The experimental uncertainty of the lattice parameters is lower than 0.003 Å. The uncertainty on pressure measurement increases from 0.06 GPa at ambient *P* to 0.27 GPa at 200 GPa.

**Figure 7:**
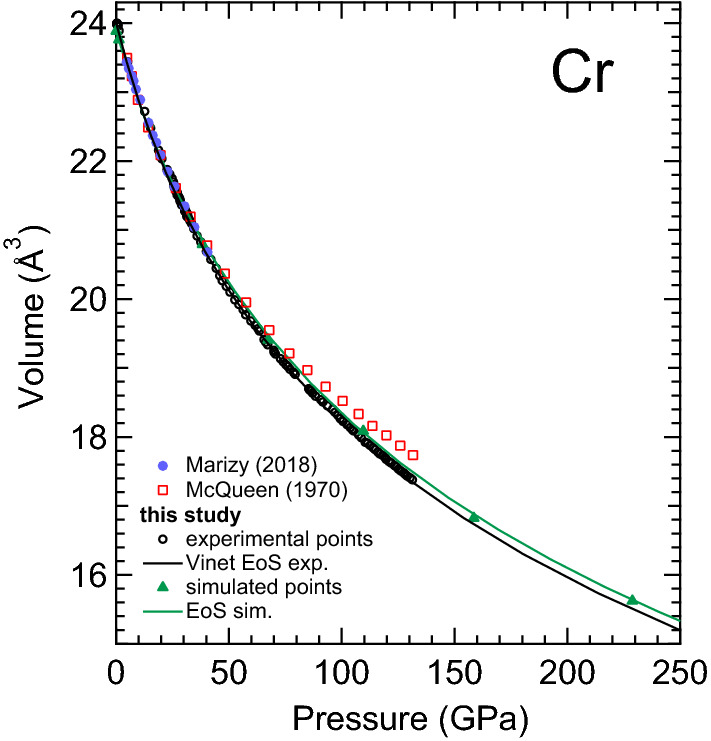
Measured and calculated unit-cell volume of Cr as a function of *P* compared with the shock data of McQueen^35^ and the XRD data of Marizy et al.^34^. The Vinet EOS obtained from present experiments and calculations are also shown.

The obtained experimental ambient *T* compression curve is reported in Fig. 7. The results are compared with the present theoretical calculations and previous experimental results obtained from static^34^ and dynamic^35^ studies. From an analysis of the data in Fig. 7 it is possible to observe how the present results are in agreement with those previously obtained in a DAC experiment performed under the same conditions^34^. Additionally, it is also clear that the data reduced from the shock experiment reported by McQueen et al. start diverging from the present data at around 33 GPa, thereby providing a volume that goes from being 0.5$\%$ higher than the present one at 33 GPa to 2$\%$ higher at 130 GPa. Such a difference, although small, is probably caused by the increasing sample temperature during shock compression.

The present compression curve has been fitted with third order Vinet and Birch–Murnaghan EoSs (BM3) using the EOSFit7 software^68^ The corresponding bulk moduli, $K_0$, and their pressure derivative, $K_0'$, and volumes, $V_0$, at ambient *P* are reported in Table 2 where they are compared with previous results. In Table 2, it can be seen that calculations slightly underestimate the volume at ambient conditions, but that they are also in excellent agreement with experiments regarding the bulk modulus and its pressure derivative. In particular, the values obtained from calculations are within the 68.3$\%$ confidence level ellipse of the experimental results. When the present bulk modulus is compared with that of previous studies it can be seen that the DAC experiments of Marizy et al.^34^ reported a bulk modulus 10 $\%$ larger than the present one; but that they also reported a smaller pressure derivative for the bulk modulus. However, their parameters fall within the 95.5$\%$ confidence level ellipse of our experimental results; i.e. we can conclude that there is a good agreement between both experiments. The small difference in the EoS parameters could be related to the use of a different pressure standard or to the different pressure ranges covered by both experiments. Shock-wave experiments also found a bulk modulus in good agreement with the present results. In contrast, the experiment by Ming et al.^31^ gives an overestimated bulk modulus. In fact, their values for $K_0$ and $K_0'$ are outside the 99.7$\%$ confidence level ellipse of our results. Their overestimation of the bulk modulus could be related to the highly non-hydrostatic conditions of their experiments which were conducted without any pressure transmitting medium. Finally, previous DFT calculations also overestimate the bulk modulus. This can be related to an overestimation of the cohesive energy due to the functionals used to describe the exchange-correlation of the energy. To conclude the discussion on the bulk modulus we compare Cr with other *bcc* transition metals. We note that when comparing group 4 and 5 (3d and 4d) transition metals, that the corresponding bulk moduli increase following the sequence: V (143) < Nb (170) < Cr (185) < Mo (210). Consequently, Cr is one of the hardest transition metals, which is also shown by the sequence of Vickers hardness: V (60 Hv) < Nb (75 Hv) < Cr (90 Hv) < Mo (120 Hv)^69^.

### Thermal equation of state

**Figure 8:**
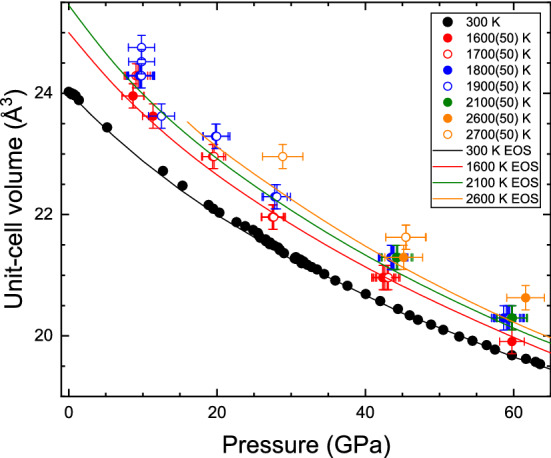
Unit-cell volume of Cr versus pressure for different temperatures. Different symbols correspond to different temperatures which are indicated in the figure. The solid lines are isotherms obtained from the P–V–T EoS determined in the present work. The 2600 K isotherm of *bcc* Cr is shown only for P > 16 GPa because at lower pressures Cr exists in the liquid state.

In Fig. 8, the measured volumetric compression is reported as a function of *P* for different *T*. The ambient *T* data points correspond to results shown in Table 2. From the *P*–*V*–*T* data shown in Fig. 8, it was possible to determine a thermal EoS using the EosFit7 package^68^. The established *P*–*V*–*T* EoS is valid up to 65 GPa. For the analysis, we used all the data included in the figure. During the fitting procedure, the third-order BM EoS generated from the *RT* compression experiment was used as the isothermal part of the *P*–*V*–*T* EoS. In addition, a Berman equation was employed as the thermal-expansion model^70^, assuming a linear variation of $K_0$ with *T*. The pressure derivative of the bulk modulus was assumed to be *P*-independent. The thermal expansion was considered to be *P*-independent and to have a linear *T*-dependence. This simple model properly describes all the available experimental results, up to 2100 K, as can be seen in Fig. 8. The model does not reproduce the results at 2600 K and 2700 K very well, probably due to the influence of anharmonic effects at such high *T*, which are not considered. The obtained parameters are d$K_0$/dT = − 0.022(9) GPa/K, volumetric thermal expansion $\alpha$ = 3.0 (5) $\times$ 10$^{-5}$ K$^{-1}$, and d$\alpha$/dT = 1.5 (5) $\times$ 10$^{-9}$ K$^{-2}$. These values are comparable to the values of the same parameters reported for *bcc* vanadium^24^, *fcc* platinum^18^ and iridium^22^, and *hcp* ruthenium^71^. This makes us confident in the *P*–*V*–*T* EoS parameters determined in the present work for chromium. These parameters can be used for calculating higher-order thermoelastic parameters; for instance $\alpha x K$ and $(dK/dT)_{V}$ = $(dK/dT)_{P}$ + $\alpha x K (dK/dP)_{T}$. From the present results, we determine that $\alpha x K$ = 0.0055 K/GPa and $(dK/dT)_{V}$ = 0.0043 GPa/K. This small value of $\alpha x K_{0}$ indicates that the thermal pressure in Cr is small, therefore Cr can be used as a pressure calibrant in *HP*–*HT* experiments. On the other hand, $(dK/dT)_{V}$ is close to zero, which is in agreement with the Swenson law^72^. The positive value obtained implies that the thermal pressure slightly increases with compression.

**Table 3:** EOS parameters of Cr measured and calculated in different experiments.

Reference	$V_0$ (Å$^3$)	$K_0$ (GPa),$K_0^{'}$	PTM	Pressure gauge	EOS	Method
This study	24.08(2)	185(1), 4.74(3)	He	W^67^	BM3	AD-XRD in DAC
This study	24.08(3)	182(1), 5.10(4)	He	W^67^	Vinet	AD-XRD in DAC
^34^	24.00(2)	200(2), 4.3(8)	He	Not specified	Vinet	AD-XRD in DAC
^31^		245(7), 5.5	None	Not specified	BM3	ED-XRD in DAC
^35^		175(3), 6.18(9)			Vinet	Shock
This study	23.9	182.4, 5.15			BM3	LDA NM
This study	23.9	182.2, 5.16			BM3	LDA PM
^34^	23.1(1)	258.0(5), 4.30(1)			Vinet	DFT

The volume $V_0$, the bulk modulus $K_0$ and its pressure derivative $K_0^{'}$ are listed. Experimental methods and EOS formulation are specified. *PTM* pressure transmitting medium, *BM3* third order Birch–Murnaghan, *ED-XRD* energy dispersive X-ray diffraction, *AD-XRD* angular dispersive X-ray diffraction.

## Conclusions

In the present study, we have determined the *P*–*T* phase diagram of chromium up to 90 GPa and 4500 K by means of LH-DAC and synchrotron-based XRD measurements. Experiments have been combined with DFT calculations up to 275 GPa and 5830 K. Experiments and calculations are in complete agreement and the obtained melting temperature as a function of *P* can be described with a Simon-Glatzel equation $T_{m}(P) = 2136K (1 + P/25.9)^{0.41}$. We have also found that Cr remains in the *bcc* phase up to 131 GPa at RT and we have determined a $P-V$ equation of state from which a bulk modulus of 182–185 GPa has been established. We have also determined the volume of Cr as a function of *P* following different isotherms, thereby obtaining a *P*–*V*–*T* equation of state and allowing Cr to be used as a pressure standard for *HP*–*HT* experiments. Finally, DFT calculations support that the determined melting curve is valid up to extreme pressures close to 1 TPa. These conclusions are supported by calculations of the radial distribution function above and below the melting temperature.

## Methods

### Experimental

Four membrane diamond anvil cells (DAC) were equipped with diamond with culets ranging from 100 to 400 $\upmu$m. The gaskets were prepared from pre-indented and spark-eroded Re foils (200 $\upmu$m original thickness). For both the high and the ambient *T* experiments the samples were taken from a $\ge$ 99$\%$ trace metal basis from Sigma Aldrich. For the laser-heating experiments, the Cr powder was initially squeezed between two diamond anvils to obtain a foil of $\sim$ 2 $\upmu$m thickness. The foil was then cut to the desired size and loaded in the DAC high pressure chamber between two disks of KBr. The KBr disks, oven dried at 250 $^{\circ }$C for a couple of hours before loading the DACs, were used as: pressure transmitting media; to insulate the sample from the diamond anvils (thermally and chemically) and as pressure gauges.

In order to maximize the hydrostatic conditions, few grains of Cr were loaded at the centre of the DAC’s high pressure chamber for the cold compression experiment. A grain of W was also added few micrometers away from the Cr sample as an X-ray standard. W was chosen due to its high X-ray scattering power and well characterized EoS, as attested to by the consistency between static, dynamic and ultrasonic measurements^73,74^. Finally, once the quality of the loading was confirmed by XRD (also used to obtain the actual V$_0$ of Cr), the high pressure chamber was filled with He pressure transmitting medium.

Both laser-heating and cold compression experiments were performed at the extreme conditions beamline I15 of Diamond Light Source^75^. The beamline’s polychromatic beam was tuned to 29.20 keV and 29.25 keV for the laser-heating and cold compression experiments, respectively. In both cases, the beam was focused down to 9 $\times$ 6 $\upmu$m$^2$ (FWHM) and a Pilatus CdTe 2M detector was used to ensure fast data collections with a good signal/noise ratio. In both cases, the sample-to-detector distance was calibrated following standard procedure from the diffraction ring of a CeO$_2$ standard.

#### Laser-heating

The *HP*–*HT* experiments were performed using the beamline’s laser-heating system^75^, following the procedure described in Anzellini et al.^18^ Before each heating ramp, the sample was brought to the target *P*, measured from the compression curve of KBr, according to the thermal EoS of Dewaele et al.^76^ In order to minimize axial thermal gradients on the sample, double sided laser-heating was performed using two 100 W Nd:YAG lasers. Both lasers were individually focused on the sample’s surfaces and tuned in order to obtain similar *T* values. Both lasers were intentionally slightly unfocused (towards the sample) and coupled together, in order to increase the FWHM of their Gaussian profile on the sample surfaces and maximise the region at uniform *T* ($\sim$ 40 $\upmu$m) probed by the X-rays. During the experiment, *T* was measured via spectral radiometry (between 450 and 950 nm), following the procedure described in Anzellini et al.^77^ *T* data were simultaneously collected from both sides of the sample and the final *T* was considered as the average between the two.

The resulting error in each *T* measurement was assumed to be the maximum value between the difference of *T* measured from the two sides of the sample, and the standard deviation of the histograms obtained from their two-colour pyrometries (Benedetti and Loubeyre^78^). The corresponding thermal *P* was obtained from the thermal EoS of Dewaele et al.^76^ under the assumption that Cr and KBr were experiencing the same *T*. Considering the present sample geometry and the corresponding axial thermal gradient, the maximum error in *P* was calculated as half the difference between the pressure obtained from the KBr at the measured *T* (when in contact with the Cr) and the one from the KBr at ambient *T* (when in contact with the diamond). Before and after each heating ramp, the alignment between the X-rays, the lasers and the *T* reading was checked following the procedure described in Anzellini et al.^75^.

The heating ramps were performed in “trigger mode”: both lasers were set to a target power; after 0.3 s a diffraction pattern and a *T* measurement were collected simultaneously; 0.3 s after the XRD collection, both laser powers were set back to zero. This procedure allowed us to minimize the interaction time between the laser and the sample (reducing the risk of possible chemical reactions) and to perform any adjustment in the optics when needed^77^. During each heating ramp, the lasers powers were increased until a diffuse signal (characteristic of liquids) was detected in the diffraction pattern or it was not possible to further increase *T*, probably due to change in the insulating conditions of the sample e.g. presence of a laser-drilled hole. Several heating ramps were performed on the same samples at different *P*–*T* conditions. In order to avoid any chemical contamination, each ramp was performed on a different region of the sample and the quality of the selected region was first checked via XRD before the actual ramp.

During the analysis procedure, good care was taken to investigate the different aspects of the experiment. *T* measurements were double checked following the procedure described in Benedetti and Loubeyre^78^. An accurate analysis of the diffraction patterns was performed to detect the appearance of the melting and to obtain structural and textural information about the sample and the insulating material. Masks were applied on a per-image basis and the images azimuthally integrated using the DIOPTAS suite^79^. KBr data were treated as powder data and a Pawley analysis was performed with the TOPAS suite^80^ using previously reported parameters as starting values. A similar analysis was originally performed for Cr. However, in order to account for the experimental thermal gradients, the thermal expansion data of Cr were obtained treating the XRD as single crystal data. A single peak of Cr (corresponding to the 110 plane) was followed during heating and integrated individually to determine the corresponding lattice parameter. Finally, the structural measurements were compared to the *T* ones so to obtain a detailed in situ and “time-resolved” analysis of the sample evolution as a function of *P* and *T*.

#### Cold compression

During the cold compression experiment, *P* inside the high-pressure chamber was estimated from the measured volume of the W X-ray standard following the calibration of Dorogokupets et al.^67^. According to the adopted pressure scale, the error in the present *P* measurements goes from 0.06 GPa at ambient *P* to 0.27 GPa at 200 GPa. During the entire compression run we tried to maintain pressure steps of the order of 0.5–1 GPa, with a stabilization period of 2 min between each XRD measurement. Diffraction data were azimuthally integrated using the DIOPTAS suite^79^, with masks applied on a per-image basis. The obtained diffraction patterns were analysed by Pawley fitting using the routines of the TOPAS software suite^80^, literature values for the lattice parameters were used as starting points for the refinement.

### Theoretical calculations

Our theoretical calculations of the equation of state and melting curve of Cr are based on density-functional theory (DFT) with the projector-augmented-wave (PAW)^81^ implementation and the generalized gradient approximation (GGA) for exchange-correlation energy, in the form known as Perdew–Burke–Ernzerhof (PBE)^82^. For these calculations the Vienna Ab initio Simulation Package (VASP) was used. The reason for choosing GGA instead of another implementation of DFT, namely, local density approximation (LDA), is that LDA has been known to not accurately describe the properties of 3d transition metals, specifically those of chromium, for which its predictions are in drastic disagreement with experiment^83,84^, and iron for which it produces an incorrect ground state: AF or nonmagnetic (NM) *bcc*^85,86^ or NM-*hcp*^87–89^ instead of FM-*bcc*. LDA also fails in assessing the strength of the magnetovolume effect. On the other hand, GGA correctly predicts the structural and magnetic phase diagrams^90^. Since the simulations of the present work were performed at *HP*–*HT* conditions, we used accurate pseudopotentials where the semi-core 4s and 4p states were treated as valence states. Specifically, Cr was modeled with 12 valence electrons per atom (3p, 3d, and 4s orbitals). We used an energy cutoff of 325 eV.

In all the runs we have verified that core overlap, if it happens at the relevant HP–HT conditions, does not affect the simulations. At P $\sim$ 120 GPa, the lattice constant of Cr, a = 2.65 Å, corresponds to interatomic separation of (a$\times \sqrt{3}/2 = 2.295$) $\sim$ 2.3 Å, consistent with the location of the first RDF peaks in Fig. 5 at $\sim$ $37\times 0.0625 = 2.3125$ Å. At this P, the ionic cores start to slightly overlap with each other: since the core radius for our pseudopotential (the largest value of RCUTs among those for each of the quantum orbitals) is 2.3 a.u., or $\sim$ 1.2 Å, the core overlap starts when interatomic distance reaches $\sim$ 2.4 Å. However, VASP handles core overlap very well: numerical errors in the calculations using VASP will remain almost negligible until the nearest neighbor distance reaches 2 $\times$ RCUT/(1.25 ± 0.05) $\sim$ 1.9 Å^91^. Hence, with these pseudopotentials, one can study systems with densities up to $\sim$ 22 g/cm$^3$, at least at low *T*. With our EoS of Cr, this density corresponds to *P* above 2 TPa.

The cold $(T=0)$ EOS was calculated using unit cells with a very dense *k*-point mesh of $50\times 50\times 50$ for high accuracy. The AF bcc-Cr was studied in the framework of fully unconstrained noncollinear magnetism developed in Ref.^90^. The theoretical melting curve of chromium was calculated via ab initio quantum molecular dynamics (QMD) simulations using the Z method implemented with VASP. The theoretical foundation of the Z method was first laid out by Belonoshko et al.^92^ and it is described in detail in Refs.^20,47,93,94^. We used a 432-atoms (6 $\times$ 6 $\times$ 6) *bcc*-Cr supercell with a single $\Gamma$-point. Full energy convergence (to ${\mathop {\sim }\limits ^{<}}1$ meV/atom) and full pressure convergence (to 0.5 GPa) were checked for both as a function of the system size and energy cutoff in each simulation. For the calculation of the melting curve the nonmagnetic structure is considered.

## Data Availability

The datasets generated during and/or analysed during the current study are available from the corresponding author on reasonable request.
